# Obesity hypoventilation syndrome

**DOI:** 10.4103/1817-1737.49411

**Published:** 2009

**Authors:** Laila Al Dabal, Ahmed S. BaHammam

**Affiliations:** *Department of Pulmonary Medicine, Rashid Hospital, Dubai Health Authority, UAE*; 1 *Sleep Disorders Center, College of Medicine, King Saud University, Riyadh, Saudi Arabia*

**Keywords:** Obesity hypoventilation syndrome, positive pressure ventilation, sleep-disordered breathing

## Abstract

Obesity is becoming a major medical concern in several parts of the world, with huge economic impacts on health- care systems, resulting mainly from increased cardiovascular risks. At the same time, obesity leads to a number of sleep-disordered breathing patterns like obstructive sleep apnea and obesity hypoventilation syndrome (OHS), leading to increased morbidity and mortality with reduced quality of life. OHS is distinct from other sleep- related breathing disorders although overlap may exist. OHS patients may have obstructive sleep apnea/hypopnea with hypercapnia and sleep hypoventilation, or an isolated sleep hypoventilation. Despite its major impact on health, this disorder is under-recognized and under-diagnosed. Available management options include aggressive weight reduction, oxygen therapy and using positive airway pressure techniques. In this review, we will go over the epidemiology, pathophysiology, presentation and diagnosis and management of OHS.

Sleep is a state of reversible unresponsiveness to the environment, in which the brain is relatively more responsive to internal than to external stimuli; and contrary to common beliefs, it is an active physiological central nervous phenomenon with cyclical changes alternating between nonrapid eye movement sleep (NREM) and rapid eye movement sleep (REM)[1]; and based on electrophysiological studies, NREM sleep is further subdivided into 4 stages (I-IV) according to recorded cortical electrical potentials. In normal young adults, sleep begins with ∼80 minutes of NREM sleep, followed by an REM period of ∼2–10 minutes. This 90-minute NREM-to-REM sleep cycle is then repeated about 3 to 6 times during the night.[2]

The control of breathing is a complex process which requires an integrated interaction between 3 major systems — the sensory system, the central controlling system and an effecter system. Input from carotid bodies, central chemoreceptors, pulmonary receptors and chest wall mechanoreceptors is conveyed to respiratory centers in the medulla, which processes the information and gives appropriate orders to the main respiratory muscles for coordinated inhalation and exhalation.[3–5] While a person is awake, behavioral and cognitive influences are also involved in breathing control; however, these are lost during sleep, and breathing takes place mainly under the control of the automatic system. The aim of this integrated circuit is to maintain PaCO_2_ and PaO_2_ in a tightly controlled state for minute-to-minute maintenance of cellular activities and metabolic demands by ensuring relatively constant rate and pattern of breathing over prolonged hours of sleep. Normal sleep can induce respiratory instability through several mechanisms. First of all, there is loss of the wakefulness drive to breathe; second, there are changes in the chemical stimuli driving breathing (PaCO_2_, PaO_2_) associated with modification of central control of breathing; and lastly, there is an increase in respiratory arousal threshold. The nocturnal reduction in minute ventilation, particularly during REM sleep, is expected to result in minimal elevation of PaCO_2_ with a corresponding drop in blood pH level, though still being maintained within physiologically accepted levels.[6–8] The concept of “loop gain” is widely used in the field of respiratory control engineering, and it has been used by several investigators in respiratory physiology to analyze the relationship between various components of the respiratory system. Respiratory physiologists repeatedly use terms like controller gain to describe central and peripheral chemoreceptors, and plant gain to describe respiratory pump; and both fall under the control of feedback loops. Having said that, one can conclude that sleep is a complex physiological process and that normal sleep architecture can be lost via several mechanisms acting at different levels of the respiratory system. This can result in a spectrum of abnormal rate or pattern of breathing manifesting as hypopnea/apnea occurring at a central or peripheral level. This is obviously an oversimplification of the underlying complex physiological processes going on during wakefulness and sleep in normal and abnormal states.

Sleep-disordered breathing (SDB) is a term used to describe a number of disorders in which there is abnormality in the frequency, pattern, upper airway resistance and/or depth of breathing during sleep. In the past 2 decades, there has been a plethora of data concerning diagnosis and management of SDB. It has been appreciated as a global health problem, as epidemiological studies have shown SDB to be affecting as many as 2% to 9% of middle-aged adults and more than 15% of older adults.[9–12] These figures mostly describe obstructive sleep apnea, as prevalence data on central sleep apnea in healthy adult population are lacking.

The most common SDB is obstructive sleep apnea/hypopnea syndrome (OSAHS), which has variable prevalence estimates according to diagnostic criteria.[13] In addition, the following disorders are included under the term SDB syndromes: Obesity hypoventilation syndrome (OHS), central sleep apnea (CSA), Cheyenne-Stokes respiration (CSR) and upper airway resistance syndrome (UARS).

## Definitions of Obesity Hypoventilation Syndrome

The World Health Organization (WHO) defines “overweight” as a body mass index (BMI) equal to or more than 25kg/m^2^, and “obesity” as a BMI equal to or more than 30kg/m^2^. Obesity is further classified into 3 classes — class 1 obesity (BMI, 30-34.9 kg/m^2^); class 2 obesity (BMI, 35-39.9 kg/m^2^) and class 3 obesity (BMI, ≥40kg/m^2^) — with increasing morbidity in proportion to increasing BMI [Table 1]. The organization estimates that by year 2015, around 2.3 billion adults will be overweight and more than 700 million will be obese.[14]

**Table 1:** Definition of overweight and obesity

Overweight: BMI 25-29.9 kg/m^2^
Class 1 obesity: BMI 30-34.9 kg/m^2^
Class 2 obesity: BMI 35-39.9 kg/m^2^
Class 3 obesity: BMI ≥ 40 kg/m^2^

The interaction between obesity and respiratory system is not straightforward, as one affects the other via several mechanisms. Excessive fat accumulation over the chest and abdomen adversely affects lung respiratory system mechanics, leading to physiological derangement and functional impairment, which can be reversed in some subjects following weight loss.[15] Of note is the point that the distribution of body fat seems to be more important than total body fat and BMI *per se.*[16,17] Obesity leads to reduction in chest wall compliance and respiratory muscle endurance with increased airway and chest wall resistance.[18] In addition, there will be loss of expiratory reserve volume accompanied in cases of morbid obesity, with reduction in total lung capacity and functional residual capacity.[19–21] Lower lung volumes with basal atelectasis predispose to localized hypoventilated areas, resulting in shunting and ventilation/perfusion mismatching.[22] The final result is hypoxemia, especially in the supine position; impaired pulmonary function, which is determined on testing; and progressively worsening disability. Obesity is also a well-recognized factor for obstructive sleep apnea and is a necessary condition for OHS; however, not all obese patients develop OHS, and the exact prevalence and pathophysiology are not fully known yet. In a recent paper studying the predictors for developing OHS in patients with OSA, it was found that 3 variables independently predicted OHS: Serum bicarbonate level (*P* < .001), apnea-hypopnea index (AHI) (*P* = .006), and lowest oxygen saturation during sleep (*P* < .001). Authors recommended measuring serum bicarbonate level in patients with severe OSA, and this should prompt clinicians to measure arterial blood gases when elevated.[23] The severity of AHI is not universally accepted as a good predictor of OHS and that AHI is not a determining factor for the development of OHS. Using the severity of AHI as a predictor of OHS may lead to significant under-recognition of this serious medical problem.[24]

OHS is defined as the combined presence of obesity (BMI,>30kg/m^2^) with awake arterial hypercapnia (PaCO_2_ >45mmHg) in the absence of other causes of hypoventilation[25] [Table 2]. Based on the American Academy of Sleep Medicine (AASM), patients with OHS may have obstructive sleep apnea/hypopnea syndrome with hypercapnia, sleep hypoventilation syndrome or a combination of sleep-related breathing disorders.[26]

**Table 2:** Diagnostic criteria for obesity hypoventilation syndrome

BMI >30 kg/m^2^
Awake arterial hypercapnia (PaCO_2_ >45 mm Hg)
Rule out other causes of hypoventilation
Polysomnography reveals sleep hypoventilation with nocturnal
hypercapnia with or without obstructive apnea/hypopnea events

## Epidemiology

The exact prevalence of OHS in the general population remains unknown, and most prevalence data describe subjects with obstructive sleep apnea, wherein its prevalence has been estimated to range from 10% to 38% in different groups.[27–31] Nowbar and colleagues reported the prevalence of OHS among hospitalized adult patients with a BMI> 35kg/m^2^ to be 31% after ruling out other causes of hypercapnia.[32] Additionally, OHS patients were reported to be heavy users of health-care resources. Berg *et al.* reported OHS patients to have higher health-care utilization several years prior to evaluation and treatment of their sleep breathing disorder.[33] There was a substantial reduction in “the number of days of hospitalization” once the diagnosis was made and treatment instituted.[33]

## Pathophysiology of OHS

**Figure 1:**
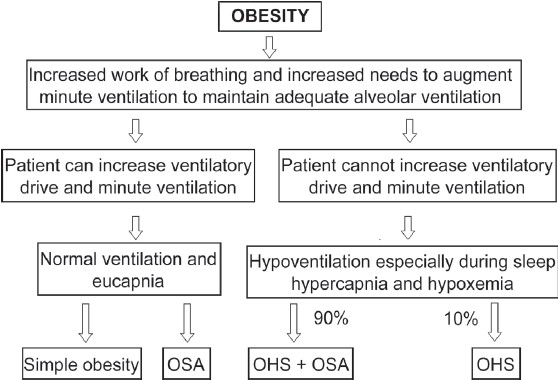
A simplified algorithm for the pathophysiology of obesity hypoventilation syndrome. OSA: Obstructive sleep apnea; OHS: Obesity hypoventilation syndrome

The pathophysiology of OHS is complex as obesity is not the only risk factor, and this may explain why the fact that some obese subjects develop OHS while others maintain normal gas exchange is not fully understood [Figure 1]. Generally, it is thought that obese subjects developing daytime hypoventilation show some form of SDB; they have reduced sensitivity to rising levels of PaCO_2_ and tend to have leptin resistance in the setting of high serum leptin level. The interaction between these factors, amongst others, leads finally to the development of the OHS picture.[34] First of all, overweight and obesity lead to a number of pulmonary and extrapulmonary complications, which can result finally in respiratory failure; albeit these cannot be taken as the sole factor inducing the development of OHS as simply, not all obese patients develop daytime hypoventilation. As highlighted earlier, obesity puts an extra mechanical load on the respiratory system, leading to its restriction and subsequent respiratory failure; however, it seems to be more complicated than this. Recent studies have shown that levels of inflammatory and pro-inflammatory markers like interleukin-6 (IL-6), tumor necrosis factor alpha (TNF alpha), interleukin-1 (IL-1), interleukin-18 (IL-18), prostaglandin E2 (PGE2) and C-reactive protein (CRP), amongst others, are elevated in obese individuals. Moreover, there is a positive correlation between IL-6 or TNF alpha plasma levels and the BMI.[35–39] There is cumulative data suggesting that obesity is characterized by chronic activation of inflammatory pathways in peripheral tissues leading to a state of insulin resistance and hypofunctioning hypothalamic C releasing hormone, which results in sleep-disordered breathing.[40,41] Along with this theory of systemic inflammatory response comes the discovery of the protein leptin as an important factor in inducing or driving OHS. Leptin is a protein produced by adipose tissues and acts on receptors in the hypothalamus to suppress appetite. It also acts on the central respiratory pathways to increase ventilation. Obese subjects have serum leptin levels significantly higher than non-obese subjects. There is growing evidence to support a role for leptin resistance in inducing or driving OHS.[42–46] In a recent paper by Campo *et al.,* a total of 245 obese subjects underwent detailed testing, and it was found that hyperleptinemia was associated with a reduction in respiratory drive and hypercapnic response, irrespective of the amount of body fat, which suggests the extension of leptin resistance to the respiratory center.[47] Various forms of SDB have been implicated in the pathogenesis of OHS, including OSA, central hypoventilation and upper airway resistance syndrome. OSA is so far the major contributor as evidenced by the facts that majority of patients with OHS have concomitant OSA (90%) and treating OSA by positive airway pressure or tracheostomy ameliorates daytime hypoventilation in most of them.[48–51] In a large group of subjects with OSA, Kawata *et al.* found that 13.7% had daytime hypercapnia. These patients had significantly higher BMI and AHI and lower PaO_2_ and vital capacity values compared with normocapnic patients, while forced expiratory volume in 1 second did not differ between the two groups. Daytime hypercapnia responded to continuous positive airway pressure (CPAP) therapy for 3 months. The authors suggested that the pathogenesis of daytime hypercapnia might be directly linked to OSA, and obesity acts as a modifier.[52] Finally, one of the important theories in the pathogenesis of hypercapnic respiratory failure in obese people relies on the observation that patients with OHS show a blunt response to hypercapnia and hypoxemia so that they fail to raise their minute ventilation in proportion to the rise in PaCO_2_ level.[53,54] In one of the earliest studies, Zwillich *et al.* confirmed reduced hypoxic and hypercapnic ventilatory drives when measured in 10 patients with OHS.[55] Gold *et al.* compared the waking pulmonary function and hypercapnic and hypoxic ventilatory responses of 35 nonhypercapnic sleep apnea patients with 17 matched non-apneic control subjects. Nonhypercapnic sleep apnea patients showed lower waking ventilatory response to hypercapnia, a higher waking PaCO_2_ level, a lower waking PaO_2_ level and a lower total lung capacity. These findings obviously mimic those seen in patients with OHS, which might suggest that there is a spectrum of ventilatory response in obese subjects being modulated by several interacting factors.[56]

## Clinical Presentation and Diagnostic Methods

The classic presentation is an obese middle-aged male (usually BMI, ≥ 35kg/m^2^) with excessive daytime sleepiness and neurocognitive function impairment. Due to the simultaneous occurrence of OSA in the majority of patients, symptoms like snoring, witnessed apneas and poor sleep quality with early morning headache and reduced performance are reported. In case of pulmonary hypertension and right-sided heart failure, patients might report symptoms like exertional dyspnea and lower limb edema. Clinical examination confirms the high BMI and might display signs of cor pulmonale and secondary pulmonary hypertension. Measuring oxygen saturation noninvasively by pulse oxymetry reveals reduced SPO_2_. An arterial blood gas taken when breathing room air confirms the presence of low PaO_2_, PaCO_2_ and a high bicarbonate level, signifying the chronic nature of the process.[15,32] Blood tests include complete blood count to rule out secondary erythrocytosis, and thyroid function test to rule out severe hypothyroidism. Electrocardiogram and transthoracic echocardiogram could demonstrate signs of right heart strain, right ventricular hypertrophy, right atrial enlargement and elevated pulmonary artery pressure.[57] Chest x-ray and, if necessarily, CT scanning of the chest should be requested to rule in or out other potential causes of hypercapnic respiratory failure. Pulmonary function testing in obese subjects typically shows mild-to-moderate restrictive defect, reduced expiratory reserve volume and reduced inspiratory/expiratory pressures.[22] Interestingly, a great percentage of these findings reverse following significant weight reduction.[58] Following this initial work-up plan, the patient is referred for full overnight polysomnography with titration studies to make the final diagnosis and start appropriate management. Persons with sleep hypoventilation syndrome may have oxygen desaturation and hypercapnia during sleep unrelated to distinct periods of obstructive apneas and hypopneas. Hypoventilation is more pronounced during REM compared to NREM sleep. Hypoventilation is defined as sustained oxygen desaturation that is not associated with obstructive apneas or hypopneas or periodic breathing.[59] If PaCO_2_ monitoring is available, it may reveal an increase of more than 10 mm Hg in PaCO_2_ level during sleep, compared with levels during wakefulness. BaHammam *et al.* have highlighted the validity of using polysomnography (PSG) in patients with sleep-disordered breathing admitted to intensive care unit with acute hypercapnic respiratory failure and have clearly shown that the use of PSG in this setting allows for an accurate diagnosis to be made early in the course of intensive care unit admission and effective intervention using noninvasive ventilation to be introduced in a timely manner. Patients who were compliant with non-invasive ventilation therapy were followed up for up to eight months after discharge by which time their arterial blood gas readings showed significant improvement.[60]

## Management of Obesity Hypoventilation Syndrome

The first step in successful management starts with making the diagnosis of OHS and referring the patient for polysomnography with titration study. The optimal management of patients with OHS requires multidisciplinary approach combining different medical and surgical subspecialties. Affected subjects require the input from internists and endocrinologists regarding their diabetes mellitus, hypertension, hyperlipidemia, heart failure and hypothyroidism therapy; a dietician for weight reduction planning; a respirologist for respiratory failure management; and a surgeon for potential bariatric surgery when needed. Moreover, patients with OHS have higher rates of intensive care admission when compared to obese patients without hypoventilation, which obviously requires an expert input from medical intensivists regarding the management of acute or chronic respiratory failure episodes. So far, no standardized guidelines exist for this crucial disorder; and in clinical practice, majority of patients are being managed by respirologists. Obviously, initial management outlines will be guided by the severity of the condition and the acuity of the presentation; however, long-term management options include the following points[61]: 1- weight reduction, 2- oxygen therapy, 3- positive pressure ventilation, 4- pharmacotherapy, 5- tracheostomy, 6- management of complications and comorbid illnesses in OHS.

## Weight Reduction

Loosing at least 10 kg of original body weight leads to improvement in pulmonary physiology and function as evidenced by improved vital capacity and forced expiratory volume.[62] Furthermore, in patients with combined OSA and OHS, weight loss leads to reduction in apnea/hypopnea index and desaturation severity.[63,64] Severe obesity is refractory to dietary management with or without behavioral or drug therapies; and in these cases, bariatric surgery has been shown to be the most effective modality of reliable and durable treatment for severe obesity. It is important to realize that weight loss cannot be used as the sole initial treatment. In practice, several mini-invasive and invasive surgical approaches exist to achieve the optimal weight in obese patients with or without OHS. Of vital importance is the fact that most of these procedures result in subjective and objective improvements in respiratory function, blood pressure and blood sugar levels.[58,65–70] The decision of referring patients for bariatric surgery is not always easy as these patients suffer at the same time from significant comorbid illnesses putting them at higher risk for general anesthesia as well as postoperative complications.[71,72] According to the guidelines issued by the National Institutes of Health, patients with a BMI greater than 35kg/m^2^ and an obesity-related comorbid condition (including OHS) or patients with a BMI greater than 40kg/m^2^ can be referred for surgical treatment.[73]

## Positive Pressure Ventilation

**Figure 2:**
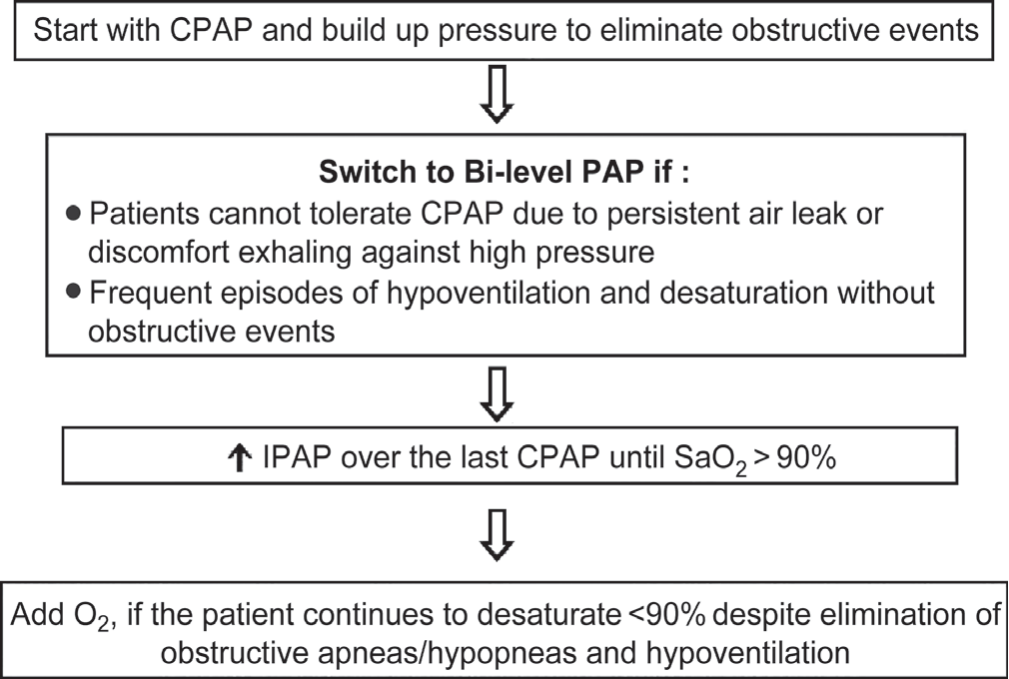
Therapeutic algorithm for positive airway pressure use in obesity hypoventilation syndrome patients. CPAP: Continuous positive airway pressure; PAP: Positive airway pressure; IPAP: Inspiratory positive airway pressure

Positive airway pressure ventilation (PAP) acutely and chronically improves gas exchange and functional status in patients with various forms of chronic respiratory failure, including those with OHS.[24,74,75] The rationale for the progressive improvement following long-term use of PAP remains speculative and is thought to act via several mechanisms leading ultimately to improved nocturnal and daytime symptoms. First of all, PAP relieves the obstructive component which is seen in the majority of patients with OHS;[76] secondly, it can effectively alter chest wall and lung mechanics in severely obese patients;[77–80] and finally, it could be acting by improving central ventilatory drive.[51,81] The first successful trial for treating OHS involved the use of CPAP therapy in 2 patients with coexisting severe sleep apnea syndrome.[82] In patients with mild OHS, CPAP and bi-level PAP (a system that allows independent adjustment of inspiratory and expiratory PAP) have been shown to be equally effective with regard to improving daytime hypercapnia.[83] Additionally, there was no difference in compliance between the two treatment modalities.[83] However, in patients with persistent hypoventilation and desaturation despite CPAP therapy, bi-level PAP should be tried.[75,84,85] It seems reasonable to start with CPAP knowing that majority of OHS patients have accompanying OSA. There are no clear guidelines on when to start or switch to bi-level PAP in patients with OHS. However, bi-level PAP should be started if the patient cannot tolerate CPAP due to persistent massive mask air leakage or has discomfort exhaling against positive pressure or if the patient has frequent episodes of hypoventilation without airway obstruction (revealed as a plateau on the inspiratory flow signal using nasal prong pressure without a thoracoabdominal paradox); or if hypercapnia persists despite being on long-term CPAP.[84–87] Pressure titration should be performed according to the protocol of American College of Chest Physicians (ACCP).[88] Oxygen supplementation should be added if the patient continues to have hypoxemia despite complete elimination of the obstructive respiratory events and hypoventilation [Figure 2].

Obviously adherence to PAP is an important modifiable predictor of improvements in ventilation and oxygenation parameters, as shown in studies looking at minimal effective duration for PAP application. In one study, investigators found that patients who used therapy for more than 4.5 hours per day experienced significant improvements in PaCO_2_ and PaO_2_ compared with less adherent patients; and another study showed that positive effects of CPAP can be seen as early as few days following its initiation.[89] Regarding long-term effects of CPAP on quality of life, Hida *et al.* found that following the use of CPAP therapy for 3 to 6 months, OHS patients had improved quality of life as evidenced by improved Short-Form (SF-36) and Epworth Sleepiness Scale (ESS); and this has been validated by other studies conducted on patients with SDB.[90–93] Follow-up studies have demonstrated significant improvement in daytime sleepiness and some physiological parameters like arterial blood gases, lung volumes and pulmonary function test parameters (forced vital capacity and total lung capacity).[48,60,76,78,94]

Average volume–assured pressure support (AVAPS) is a new hybrid mode that has the advantages of the combination of pressure-limited and volume-limited modes of ventilation into one ventilation mode to ensure a more consistent tidal volume and hence minute volume. Storre *et al.* have recently validated its use in a group of 10 patients with stable OHS. OHS patients not responding to CPAP therapy were randomly assigned to receive bi-level PAP or bi-level PAP plus AVAPS therapy in a crossover design. The main finding was that sleep quality and gas exchange substantially improved during nocturnal bi-level PAP therapy compared to baseline, but patients remained hypercapnic overnight even after 6 weeks of home mechanical ventilation following the careful establishment of bi-level PAP therapy in hospital. The addition of AVAPS to bi-level PAP therapy resulted in a significant decrease in PaCO_2_; however, this was without providing further clinical benefits regarding sleep quality and quality of life in their cohort of patients.[95]

Based on the above-mentioned literature, it becomes conceivable that various forms of PAP are effective in providing short-term and long-term benefits upon adequate utilization in patients with OHS with or without OSA, and larger studies are needed in this group of patients to decide about the optimal mode of management.

## Oxygen Therapy

OHS is characterized by prolonged attacks of sustained hypoxemia during sleep, in addition to daytime hypoxemia. Oxygen supplementation might be beneficial in patients with persistent hypoxemia despite the relief of upper airway obstruction by positive airway pressure to prevent the long-term effects of hypoxemia on pulmonary vasculature and other vital organs. It should be kept in mind, however, that treatment with oxygen alone is inadequate and not recommended as it does not reverse hypoventilation or airway obstruction on its own.[96–98]

## Tracheostomy

Before the advent of PAP modalities, tracheostomy was used more commonly in the sixties for patients with obstructive sleep apnea, and it could still be a valid choice in patients with severe upper airway obstruction who are totally intolerant to PAP and when no other options are applicable. In the few reported cases, tracheostomy showed positive impact in terms of patients' symptoms, improved respiratory drive to hypercapnia and reversal of hypoventilation.[99–102]

## Pharmacotherapy

Few drugs known for their respiratory stimulant effects, like progesterone, acetazolmide, almitrine and aminophylline, have been tried in patients with sleep apnea syndromes; however, the two most widely quoted drugs when dealing with OHS patients are medroxyprogesterone and acetazolmide.[103–106] Medroxyprogesterone acetate, a synthetic progesterone derivative which effectively stimulates breathing, has been used for long time for managing patients with OHS, with documented positive effects resulting mainly in increasing the ventilatory response to hypercapnia, which ultimately leads to improved ventilation with a drop in PaCO_2_ and a rise in PaO_2_.[107–109] Recent work by Saaresranta *et al.* on a group of postmenopausal females with respiratory failure — where study end points were PaCO_2_ level, leptin level and neuropeptide Y level — has shown that after 14 days of medroxyprogesterone acetate therapy, there was significant improvement in ventilation and reduction in PaCO_2_ without altering serum leptin or neuropeptide Y levels.[110] Obviously this approach of hormonal manipulation is not without side effects, and the risk of inducing a hypercoagulable state should always be considered, particularly in this group of patients who already have underlying predisposing factors like obesity, reduced mobility and heart failure.[111,112] The carbonic anhydrase inhibitor acetazolmide is a weak diuretic causing metabolic acidosis. When used in patients with OHS, it leads to reduction in serum bicarbonate level, which drives a mild metabolic acidosis leading to a rise in minute ventilation, which in turn leads to a reduction in PaCO_2_ level.[113,114] Currently no strong recommendations can be made about the use of both agents as no data exist on long-term safety.

## Management of Comorbid Illnesses and Complications in OHS

Obesity is a systemic process in which multiple organ systems are involved, among which the cardiovascular system, respiratory system and metabolic system seem to be affected mostly.[115,116] Hence it becomes imperative to approach the obese patient with a multidisciplinary view and try to optimize pharmacological and nonpharmacological therapies for each affected system. For example, blood pressure, blood sugar and lipid profile should ideally be maintained within normal limits. Any concomitant degree of systolic or diastolic heart failure should be aggressively managed to avoid any further compromise of the cardiopulmonary system. In addition, a search for significant complications like secondary erythrocytosis and secondary pulmonary hypertension should be carried out and appropriate interventions implemented as recommended. Secondary erythrocytosis develops to improve tissue oxygenation in the setting of chronic hypoxemia; however, this could be limited by the development of hyperviscosity.[117]

Phlebotomy is a valid option in adult patients with symptomatic hyperviscosity, but so far this has not been studied in patients with OHS.[118] Few papers shed light on the role of activated rennin-angiotensin system and the development of secondary erythropoiesis implicating a potential benefit of blocking the system with angiotensin converting enzyme inhibitor or angiotensin II receptor blockers leading to better management of polycythemia in these patients.[119,120]

In summary, obesity is a major public health problem all over the world and has detrimental effects on the economics of health- care systems at different levels. The best tool in managing this problem would be in targeting school children and preventing its evolution. However, when complications arise, there should be a high index of suspicion and pre- planned diagnostic and therapeutic approaches. Currently, the best available options for treating OHS patients are weight reduction and positive airway pressure ventilation.
